# Uncovering the silent public health threat: nasal carriers of linezolid-resistant, vancomycin-intermediate and mupirocin-resistant MRSA among healthcare workers in a tertiary care hospital in Central India

**DOI:** 10.3205/dgkh000547

**Published:** 2025-05-08

**Authors:** Neha S. Bawankar, Prashant P. Meshram, Riya John, Dilip S. Gedam, Swati M. Bhise, Nanda A. Ranshoor, Seema R. Bais

**Affiliations:** 1 Department of Microbiology, Indira Gandhi Government Medical College, Nagpur, Maharashtra, India; 2 GNM Infection Control Nurse, Indira Gandhi Government Medical College, Nagpur, Maharashtra, India

**Keywords:** Multidrug-resistant staphylococci nasal carriers, healthcare workers, MRSA, mupirocin-resistant MRSA, linezolid-resistant S. aureus, vancomycin intermediate-resistant S. aureus, biofilm producer, hand hygiene

## Abstract

**Introduction:**

Healthcare-associated infections caused by multidrug-resistant (MDR) *Staphylococcus* strains pose a significant challenge. Healthcare workers (HCWs) are potential vectors in transmitting these strains. This study assessed the prevalence of nasal carriage of staphylococci among HCWs.

**Methods:**

This prospective cohort study was conducted from March to June 2024 at a tertiary care hospital in Central India. Nasal swabs from 178 HCWs were collected and screened for methicillin-sensitive *S. aureus* (MSSA), methicillin-resistant *S. aureus* (MRSA), methicillin-sensitive coagulase-negative staphylococci (MS-CONS), and methicillin-resistant CONS (MR-CONS) using standard microbiological methods. Antimicrobial susceptibility and biofilm production were evaluated.

**Results:**

Of 178 HCWs, 61.8% were *Staphylococcus* carriers, including 36% MRSA. High MRSA carriage was observed in junior residents, interns, and nursing assistants, particularly in the surgical department. Furthermore, the notifiable carriage rate was observed among HCWs who did not consistently adhere to hand-washing practices and/or frequently picked their noses, and those regularly involved in patients’ wound care. All MRSA and MR-CONS were MDR, while 30% of MSSA and 45.5% of MS-CONS were MDR. No vancomycin resistance was detected, but 12.5% of MRSA showed intermediate resistance to vancomycin (VISA). Linezolid resistance was observed in 10% and 37.5% of MRSA (LRSA) and CONS, respectively. Biofilm production was noted in 72.7% of isolates.

**Conclusion:**

The high prevalence of nasal carriers of MRSA and MDR staphylococci strains and the emergence of VISA and linezolid-resistant staphylococci underscores the need for stringent infection control and antimicrobial stewardship measures in healthcare settings. Regular screening and decolonization protocols for HCWs are critical in preventing the spread of resistant pathogens.

## Zusammenfassung

**Einleitung:**

Nosokomiale Infektionen, die durch multiresistente (MDR) Staphylokokken-Stämme verursacht werden, stellen eine große Herausforderung dar. Beschäftigte im Gesundheitswesen (HCWs) sind potenzielle Vektoren bei der Übertragung dieser Stämme. In dieser Studie wurde die Prävalenz von Staphylokokken im V. nasi bei HCWs untersucht.

**Methode:**

Die prospektive Kohortenstudie wurde von März bis Juni 2024 in einem Krankenhaus der Tertiärversorgung in Zentralindien durchgeführt. Nasenabstriche von 178 HCWs wurden entnommen und unter Verwendung mikrobiologischer Standardmethoden auf Methicillin-empfindliche *S. aureus* (MSSA), Methicillin-resistente *S. aureus* (MRSA), Methicillin-empfindliche Coagulase-negative Staphylokokken (MS-CONS) und Methicillin-resistente CONS (MR-CONS) untersucht. Die antimikrobielle Empfindlichkeit und die Biofilmproduktion wurden bewertet.

**Ergebnisse:**

Von 178 HCWs waren 61,8% Staphylokokken-Träger, davon 36% MRSA. Eine hohe MRSA Prävalenz wurde bei jüngeren Assistenzärzten, Praktikanten und Pflegehelfern beobachtet, insbesondere in der chirurgischen Abteilung. Darüber hinaus wurde die höchste Prävalenz bei HCWs beobachtet, die sich nicht konsequent die Hände wuschen, häufig in der Nase bohrten und regelmäßig an der Wundversorgung beteiligt waren. Alle MRSA und MR-CONS waren MDR, während 30% der MSSA und 45, 5% der MS-CONS MDR waren. Es wurde keine Vancomycin-Resistenz festgestellt, aber 12,5 % der MRSA waren intermediär resistent gegen Vancomycin (VISA). Eine Linezolidenresistenz wurde bei 10 % bzw. 37,5 % der MRSA (LRSA) bzw. CONS beobachtet. Die Produktion von Biofilmen wurde bei 72,7 % der Isolate festgestellt.

**Schlussfolgerung:**

Die hohe Prävalenz nasaler Träger von MRSA- und MDR-Staphylokokkenstämmen und das Auftreten von VISA- und Linezolid-resistenten Staphylokokken unterstreicht die Notwendigkeit einer stringenten Infektionskontrolle und von antimikrobiellen Stewardship-Maßnahmen im Gesundheitswesen. Regelmäßige Screening- und Dekolonisierungsprotokolle für HCWs sind entscheidend, um die Ausbreitung resistenter Krankheitserreger zu verhindern.

## Introduction

Gram-positive infections have become a serious problem, especially in the nosocomial setting, and the emergence of multidrug-resistant pathogens further complicates the effective treatment of these infections [1]. Among Gram-positive pathogens, Methicillin-resistant *Staphylococcus aureus* (MRSA) seriously threatens healthcare facilities with increased morbidity and mortality, increasing healthcare expenditures [2]. 

Though infrequent, postoperative MRSA surgical site infection (SSI) is associated with substantial morbidity and economic burden [3]. In the context of healthcare facilities, the colonization of healthcare workers (HCWs) by *S. aureus* or MRSA presents a substantial challenge, as it can lead to transmission of the pathogen to patients due to substandard infection control practices. Previous research has underscored the significance of MRSA screening in mitigating the risk of postoperative MRSA SSI [4], [5].

According to clinical practice guidelines, routine screening HCWs for MRSA is not recommended. However, it is suggested that screening for MRSA could be beneficial in certain situations. These include

1. if transmission of MRSA continues on a ward, despite the implementation of active control measures, or if there are unusual epidemiological aspects of an outbreak;
2. it may also be beneficial if there is evidence suggesting that MRSA carriage persists among HCWs [2]. If new MRSA carriers have been found among patients in a ward, then HCWs with skin lesions should be identified and screened [6].

The analysis of the antimicrobial susceptibility data of our institute from August 2023 to March 2024 using WHO-NET software showed a very high prevalence (~80%) of MRSA infection at our institute, especially among post-operative patients. *S. aureus* accounted for >50% of SSI. This high prevalence not only poses a significant risk to patient safety but also increases the economic burden on our healthcare system. Therefore, this study aimed to screen HCWs in our hospital for methicillin-sensitive *S. aureus* (MSSA), MRSA, coagulase-negative S. aureus (CONS), and methicillin-resistant CONS (MR-CONS) carriers, and offer decolonization treatment to the carriers. This will help to strengthen hospital-associated infection (HAI) prevention and control practices in our hospital.

## Materials and methods

### Study design 

This prospective cohort study was conducted from March 2024 to June 2024 in an 822-bed tertiary care hospital in Central India. The study was approved by the Institutional Ethics Committee (approval number IEC/2128-29/2024). 

### Study population 

The study included HCWs involved in primary patient care and all essential medical activities, including senior doctors, residents, nurses, nursing assistants, and sweepers. Only the HCWs who had no history of using nasal mupirocin ointment/spray or had not taken a chlorhexidine bath in the last month were included in the study. The required questionnaires were asked to analyse the associated factors with carrier state. Participation in the survey was voluntary, respecting the autonomy and choice of all healthcare workers. 

### Sampling technique and identification of bacterial strains 

Screening samples were taken from the nostrils by rotating a single sterile cotton swab (STERISTICK^®^) pre-moistened with sterile distilled water 2–3 times around the inside of the nostril, using the same swab for both nostrils. Samples were transported to the laboratory using Stuart’s transport media. Enrichment was done by inoculating and incubating the sample overnight at 35°C in 10% salt-cooked meat broth (Hi-Media, Mumbai, Maharashtra), followed by subculturing onto blood agar. Identification and speciation of staphylococci were done using conventional microbiological methods, including colony morphology, Gram staining, and slide coagulase test, and confirmed by the tube coagulase test and other biochemical panels. Routine antimicrobial susceptibility testing for penicillin-G (10 units), cefoxitin (30 µg), clindamycin (2 µg), erythromycin (15 µg), doxycycline (30 µg), tetracycline (30 µg), levofloxacin (5 µg), gentamicin, ciprofloxacin (5 µg), chloramphenicol (30 µg), trimethoprim-sulfamethoxazole (1.25/23.75 µg), mupirocin (10 µg and 200 µg) and linezolid (30 µg) was performed using commercially available antimicrobial disks on Muller-Hinton agar (Hi-Media, Mumbai, Maharashtra) employing Kirby-Bauer’s disk diffusion method. MRSA and MR-CONS were identified based on the zone size of a cefoxitin disk. Vancomycin MIC was determined using the agar dilution method. Results were interpreted according to CSLI-2023 guidelines. *S. aureus* ATCC^®^ 25923 and *S. aureus* ATCC^®^ 29213 were used as quality control strains for the disk diffusion and MIC methods, respectively.

The study excluded HCWs not in direct contact with patients or *S. pseudointermedius, S. schleiferi*, and Gram-negative bacterial isolates.

### Biofilm formation assay (microtiter plate method) 

Four µl of bacterial overnight culture were inoculated into 1 ml of tryptic soy broth containing 0.25% glucose. Subsequently, a 96-well microtiter plate was inoculated with 200 µl of the bacterial suspension, and the plate was then subjected to a 3-day incubation period at 37°C to allow biofilm formation. Following incubation, the liquid phase was aspirated, the wells were washed with phosphate-buffered saline, and the plate was stained using a 1% crystal violet solution for 15–30 min. The excess stain was washed off, and the plate was air dried. The biofilm-bound stain was then solubilized using 95% ethanol, and the plate’s optical density (OD) of 490 was measured using a microplate reader (LisaScan EM, Erba, Mannheim, India). The OD value of each well was subtracted from that of the negative control (NG) well. The biofilm producer interpretation was based on OD values compared to NC as ≤0.5=non-producer, 0.5–1=moderate, and >1=strong producer. 

### Statistical analysis 

The research findings were subjected to statistical analysis using descriptive statistics and the Chi-squared test. A p-value<0.05 indicated statistical significance. The risk factor analysis of MRSA colonization was done utilizing SPSS version 22.

### Decolonization treatment and follow-up plan for MRSA carriers 

HCWs carrying *S. aureus*, MRSA, CONS, or MR-CONS were informed personally to maintain confidentiality. They were offered a decolonization course of 2% mupirocin nasal ointment and 4% chlorhexidine gluconate for daily bathing or showering for five days to eliminate the bacteria from their body. They were screened after one week of complete treatment and at the 4^th^, 8^th^, and 12^th^ weeks. If they returned a positive result during this time, repeated decolonization ≥2 positive consecutive cultures of nasal swabs were found with the same staphylococcal species, the carrier was categorized as a persistent carrier, and those who were positive <2 times were referred to as intermittent carriers.

## Results

### Prevalence of staphylococcus carriage

Among 178 participants enrolled in the study, 110 (61.8%) were identified as *Staphylococcus* carriers. The prevalence of MSSA, MRSA, MS-CONS, and MR-CONS was 16.9% (30/178), 36.0% (64/178), 6.2% (11/178), and 2.8% (5/178), respectively. All carriers were asymptomatic. Maximum carriers were in the intermittent category, while two were persistent carriers harboring MRSA (Table 1 (Tab. 1)). 

### Socio-demographic characteristics and risk factors of carriers 

Table 2 (Tab. 2) presents the distribution of *S. aureus* and CONS carriers across different demographic and risk factors. Although the study found the highest number of *S. aureus* (MRSA and MSSA) carriers in the 20–29 age group with a preponderance of females, no significant association was found between age group and carrier rate (p>0.05). None of the comorbid conditions were significantly associated with the nasal carriage rate of *Staphylococcus*.

Interestingly, the notifiable association between the distribution of different *Staphylococcus* phenotypes was found across various departments, settings, and professions. Most *Staphylococcus* carriers, 80.9% (89/110), were from surgical departments (obstetrics-gynecology [OBGY], surgery, ophthalmology, orthopedic, and ENT). The MRSA carrier rate was highest in OBGY (37.5%), followed by surgery (20.3%). Further, the carrier rate was higher among HCWs working on wards than in intensive-care units (ICUs). The MRSA carrier rate was exceptionally high among junior residents-1, interns, and nursing assistants.

### Association between habits and MRSA carriage 

The study found a strong association of staphylococcal carriage with insufficient handwashing, nose-picking habits, and wound-dressing involvement (p<0.05) (Table 3 (Tab. 3)). 

### Antimicrobial susceptibility of MSSA, MRSA and CONS

All the MRSA and MR-CONS were multidrug-resistant (MDR), while 30% (9/30) MSSA and 45. 5% (5/11) MS-CONS were MDR. None of the strains was extensively drug resistant or pan-drug resistant.

**Figure 1:**
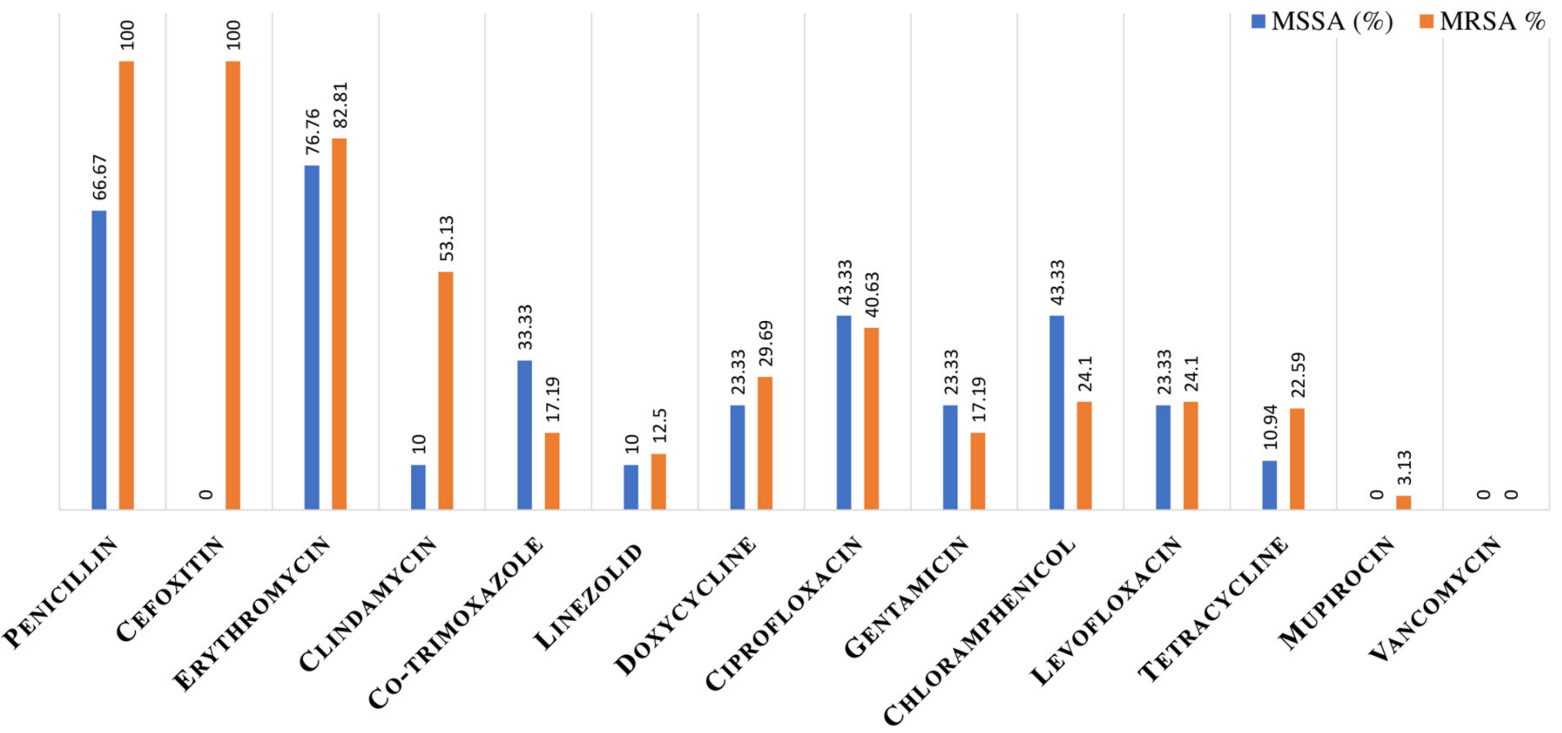
Antimicrobial susceptibility of methicillin-sensitive *S. aureus* (MSSA) and methicillin-resistant *S. aureus* (MRSA) isolated from nostrils of healthcare workers

Penicillin exhibited a resistance rate of 66.7% in MSSA. Erythromycin resistance was observed at high levels in MSSA (76.86%) and MRSA (82.8%). In MSSA, clindamycin and tetracycline displayed a notably lower resistance rate (10% and 10.9%, respectively) than did MRSA (53.1% and 22.6%, respectively). Among clindamycin-resistant isolates, constitutive and inducible resistance was found in 6.7% and 3.3% of MSSA and 37.5% and 15.3% of MRSA, respectively. Furthermore, trimethoprim-sulfamethoxazole, linezolid, and gentamicin showed higher resistance rates in MSSA (33.3%, 10%, and 23.3%, respectively) than in MRSA (17.2%, 12.5%, and 17.2%, respectively). Ciprofloxacin resistance was similar in both groups, approximately 43.3% in MSSA and 40.6% in MRSA. The resistance to chloramphenicol and levofloxacin was slightly higher in MSSA (43.3% and 23.3%, respectively) compared to MRSA (24.1%) (Figure 1 (Fig. 1)).

**Figure 2:**
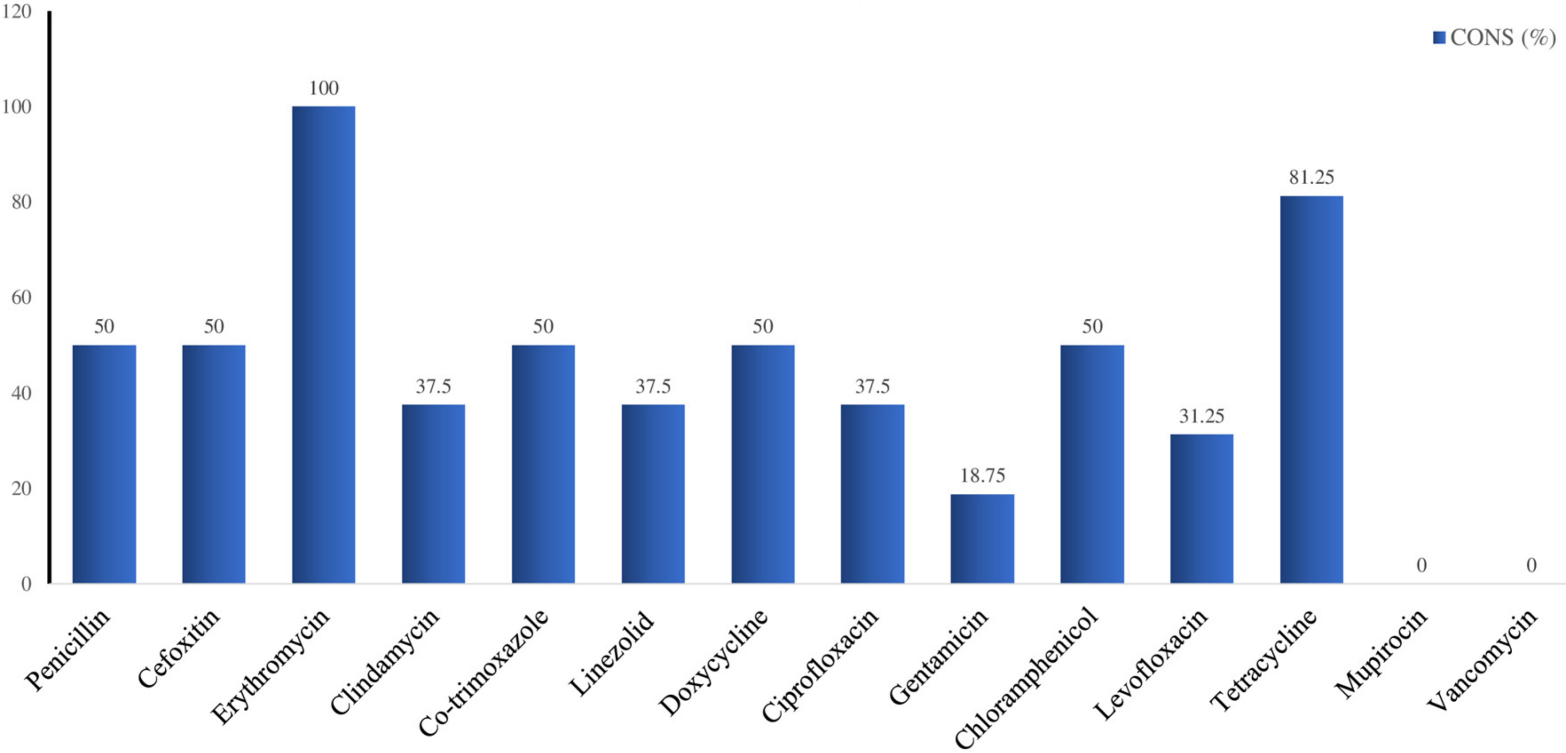
Antimicrobial susceptibility of coagulase-negative *S. aureus* (CONS) isolated from nostrils of healthcare workers

All the CONS isolates were resistant to erythromycin, and 50% were resistant to penicillin-G, cefoxitin, trimethoprim-sulfamethoxazole, doxycycline, and chloramphenicol. Moderate resistance (37.5%) was observed against clindamycin, linezolid, and ciprofloxacin. A lower resistance rate (18.8%) was observed against gentamicin. Tetracycline had a high resistance rate of 81.3%. (Figure 2 (Fig. 2)).

Notably, vancomycin and mupirocin resistance was not found in all the studied strains. Nevertheless, high-level mupirocin resistance was seen in MRSA strains isolated in two persistent carriers of MRSA in the third screening round after 14 days of decolonization treatment. However, eight strains (12.5%) of MRSA showed intermediate resistance to vancomycin (VISA), of which two had an MIC of 4 µg/mL and six had an MIC of 8 µg/mL. Of 64 MRSA, thirty strains (46.88%) had MIC ≥1.5 µg/mL. 

### Biofilm-forming strains 

Among 110 isolates tested, 30 (25 MSSA, 5 MS-CONS) were non-biofilm producers, 77 (5 MSSA, 62 MRSA, 6 MS-CONS, and 4 MR-CONS) were moderate biofilm producers, and 3 (2 MRSA, 1 MR-CONS) were strong biofilm producers. 

## Discussion

The Centers for Disease Control and Prevention have now recognized MRSA as a serious public health threat. Its worldwide rates have increased dramatically during the last decades [7]. Furthermore, CONS form a large group of skin microbiota that play an increasingly important role, especially in HAI and infections in immunocompromised patients [8]. Although it has long been considered a contaminant, the rise of antimicrobial resistance in recent years has dramatically impacted HAIs caused by CONS [8], [9].

Even though *S. aureus* can be cultured from multiple sites of carriers’ mucosal surfaces and skin, the ecological niches of *S. aureus* strains are the anterior nares. Extensive research has demonstrated that the nares consistently serve as the primary site for isolating this organism. Notably, targeted topical treatment of the nares to eliminate nasal carriage typically results in the organism’s clearance from other body sites in most cases [10]. HCWs play a crucial role in the spread of HAI by carrying resistant strains of bacteria in hospitals. Therefore, it is essential to detect colonization of MRSA and MR-CONS among HCWs, particularly those working in surgical units, as they can potentially transmit infections to their immunocompromised patients.

In the present study, the nasal carriage rate of staphylococci was 61.8%, with MRSA accounting for 36%, indicating significant colonization among HCWs. In other parts of India and South Asia, the prevalence of MRSA nasal carriers among HCWs was reported as ranging from 0.7% to 36.16% [3], [11]. Comparatively low prevalences have been reported in Western countries [12], [13]. Only a few (2.8%) of the HCWs in our study were MR-CONS carriers, compared to studies from other parts of India [14], [15]. However, not only MRSA and MR-CONS were MDR, but also a significant proportion of MSSA and MS-CONS strains were. This emphasizes the need for continued monitoring and effective antimicrobial management to tackle the issue of multidrug resistance in staphylococcal strains, regardless of methicillin resistance.

The OBGY department had the highest rate of carriers, followed by other surgical departments, with most carriers being junior resident-1s, interns, and nursing assistants. The higher carrier rate among these HCWs may be due to frequent contact with patients, contaminated objects, and the hospital environment [16]. Some studies reported the highest carrier rate among nurses [17], [18], [19]. Furthermore, the notifiable carriage rate was observed among HCWs who did not consistently adhere to hand-washing practices and/or frequently picked their noses, and those regularly involved in patients’ wound care. These findings reflect the significant role of HCWs in transmitting MDR staphylococci strains, particularly from patients to HCWs and back to patients. Rai et al. [20] also reported a significant association between nose-picking, handwashing habits, and MRSA carriage. However, the ICUs (including neonatal, pediatric, medicine, and surgical ICUs) had the lowest prevalence of carriers compared to the wards. This discrepancy may be attributed to HCWs’ stringent adherence to wearing personnel protective equipment and following hand hygiene protocols during patient care within the ICU, which helps break the vicious cycle of staphylococcal transmission. 

Although no vancomycin-resistant *S. aureus* strains (VRSA) were detected, 12.5% were found to be VISA, and 46.9% had a MIC ≥1.5 µg/mL, raising concerns about the potential emergence of vancomycin resistance, the primary anti-MRSA treatment, in developing countries. A study (2020–2023) from another tertiary care center in Central India also reported 9.5% VISA in clinical isolates of MRSA [7]. Furthermore, linezolid-resistant *S. aureus* (LRSA) strains in the study point toward an upcoming anti-MRSA crisis. Despite the fact that the use of mupirocin is restricted for infection control, there are increasing reports of mupirocin-resistant strains across the globe [21], [22]. The absence of low-level mupirocin resistance in our study was reassuring; however, detecting high-level mupirocin resistance in two persistent MRSA carriers suggests a potential for resistance development with repeated use, urging caution and vigilance. These carriers’ other sites (hands, axilla, throat, and groin) were screened. One had a history of recurrent folliculitis (groin), and the other one also carried the MRSA strain with the same sensitivity pattern in the axillae. Both were treated with repeated decolonization and systemic therapy according to strain sensitivity. 

In the study, MDR phenotypes were more prevalent among CONS than *S. aureus*. Infection with these MDR strains increases the likelihood of treatment failure, but also facilitates the transmission of this resistance to *S. aureus*, thereby presenting a significant challenge for healthcare practitioners. This emphasizes the significance of screening nasal carriage of HCWs for these phenotypes of staphylococci, as these may serve as forerunners of MRSA/MR-CONS/VRSA. The higher MDR strains in the study could be due to the misuse, excessive use, and irrational prescriptions of these medications in our hospitals and the community. 

Most (70%, 77/110) of the strains in the study were moderate- and 2.7% (3/110) were strong biofilm producers. This property helps them survive longer on the nasal mucosa and surfaces of devices (potential sources of device-associated HAI). Therefore, MSSA and CONS colonizers should be screened and decolonized, especially in ICU settings. Additionally, regular screening of HCWs who are carriers of MRSA provides insight into the effectiveness of hospital infection control measures and offers a basis for ameliorating any existing flaws in the methods. 

This study observed that age, gender, or comorbid conditions in HCWs were not significant determinants of staphylococcal carriage. Contrary to these findings, Shibabaw et al. [17] reported a high prevalence of MRSA carriers among males compared to female HCWs. Duong et al. [3] reported a high prevalence among HCWs with comorbidities. 

### Limitations

The limitations of this study include the reliance on voluntary participation of HCWs, which may introduce selection bias. The study also focused on HCWs in direct contact with patients, excluding other hospital staff who may also play a role in pathogen transmission. The study’s timeframe of four months may not capture seasonal variations in MRSA carriage rates among HCWs. Genotyping of the strains could not be done due to limited resources. Furthermore, the study’s focus on a single tertiary care hospital in Nagpur, India, may limit the generalizability of the findings to other healthcare settings with different demographics and infection control practices.

## Conclusions

This study underscores the significant burden of *Staphylococcus* carriage, particularly MRSA, among HCWs in this region. Notably, a higher rate of MDR strains of CONS and MSSA warrants screening of nasal carriers for these phenotypes. The isolation of VISA and LRSA in carriers points towards an upcoming anti-MRSA crisis and mirrors the existence of such strains in the hospital environment. Consequently, robust antimicrobial stewardship initiatives and strict adherence to infection control protocols are imperative to combat the escalating menace of antibiotic resistance in staphylococci. Regular screening and decolonization protocols for HCWs are critical in preventing the spread of resistant pathogens.

## Notes

### Competing interests

The authors declare that they have no competing interests.

### Ethical approval 

The study was approved by the Institutional Ethics Committee (approval number IEC/2128-29/2024). 

### Funding

None. 

### Authors’ ORCIDs

- Bawankar NS: https://orcid.org/0000-0003-2504-0941
- Gedam DS: https://orcid.org/0009-0009-0862-0830
- Bhise SM: https://orcid.org/0009-0008-2109-4145

## Figures and Tables

**Table 1:**
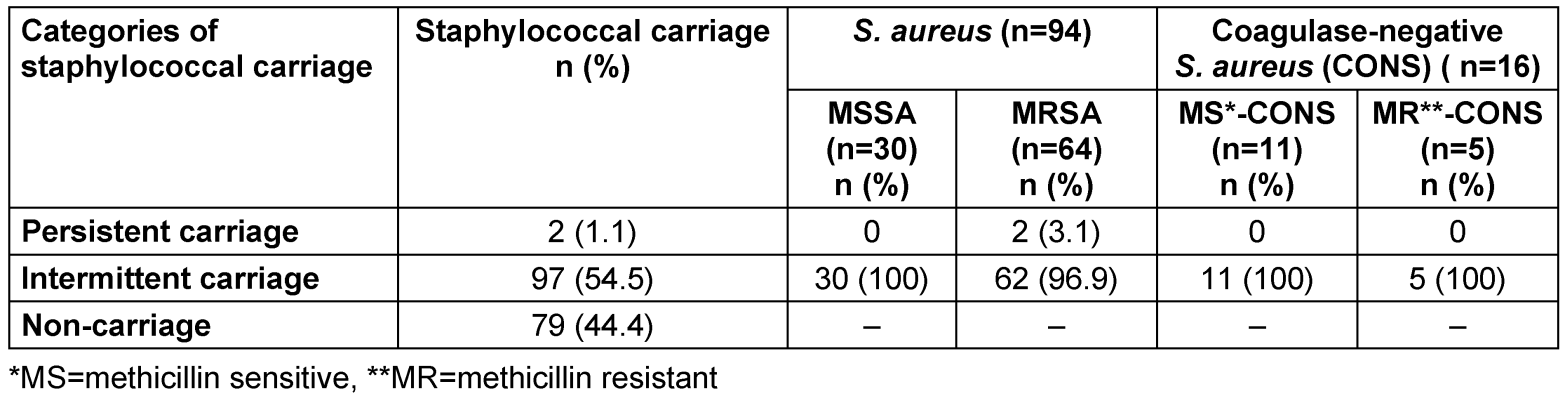
Patterns of staphylococcal carriage among healthcare workers

**Table 2:**
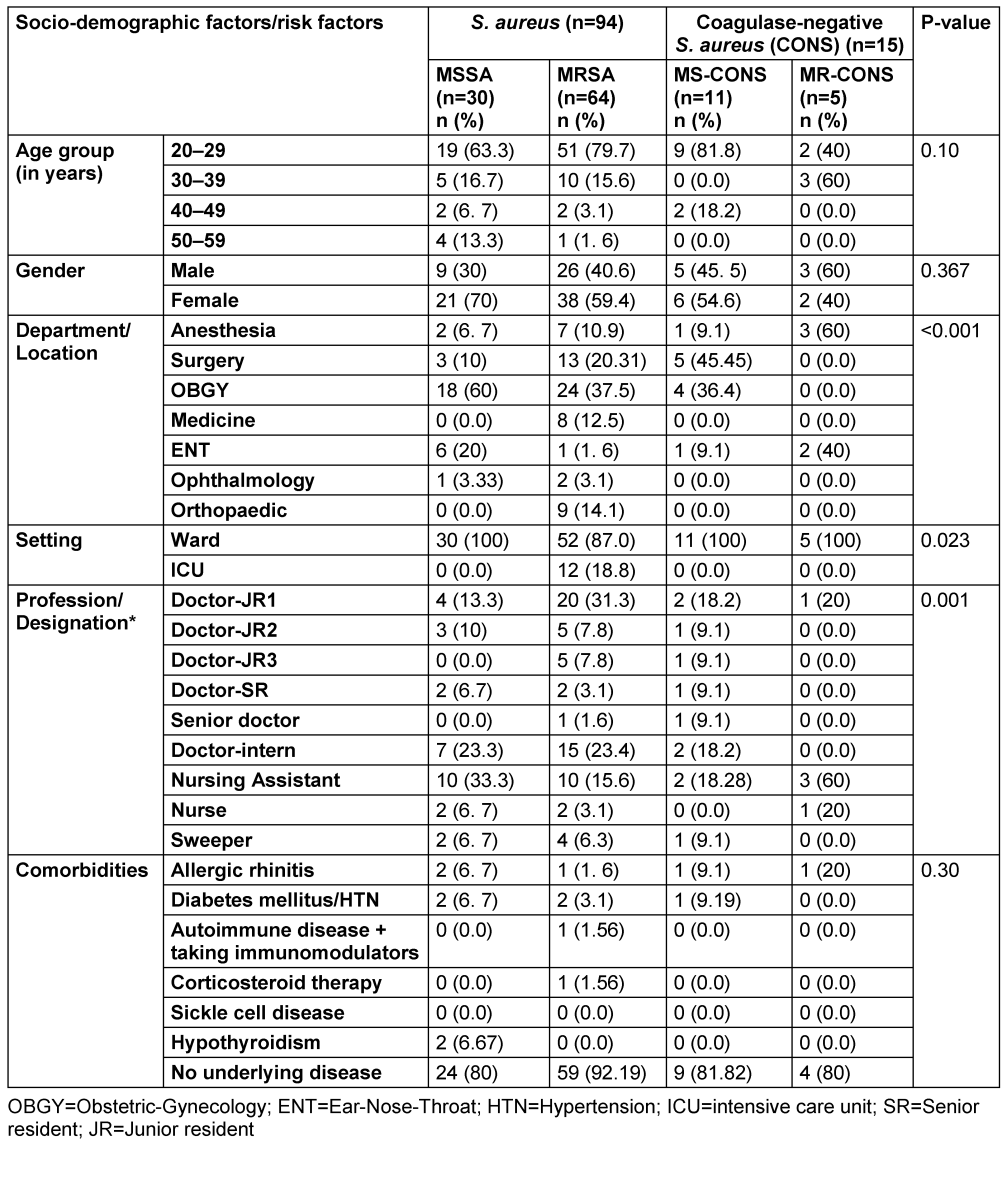
Socio-demographic factors and risk factors associated with different *Staphylococcus* phenotypes of nasal carriage

**Table 3:**
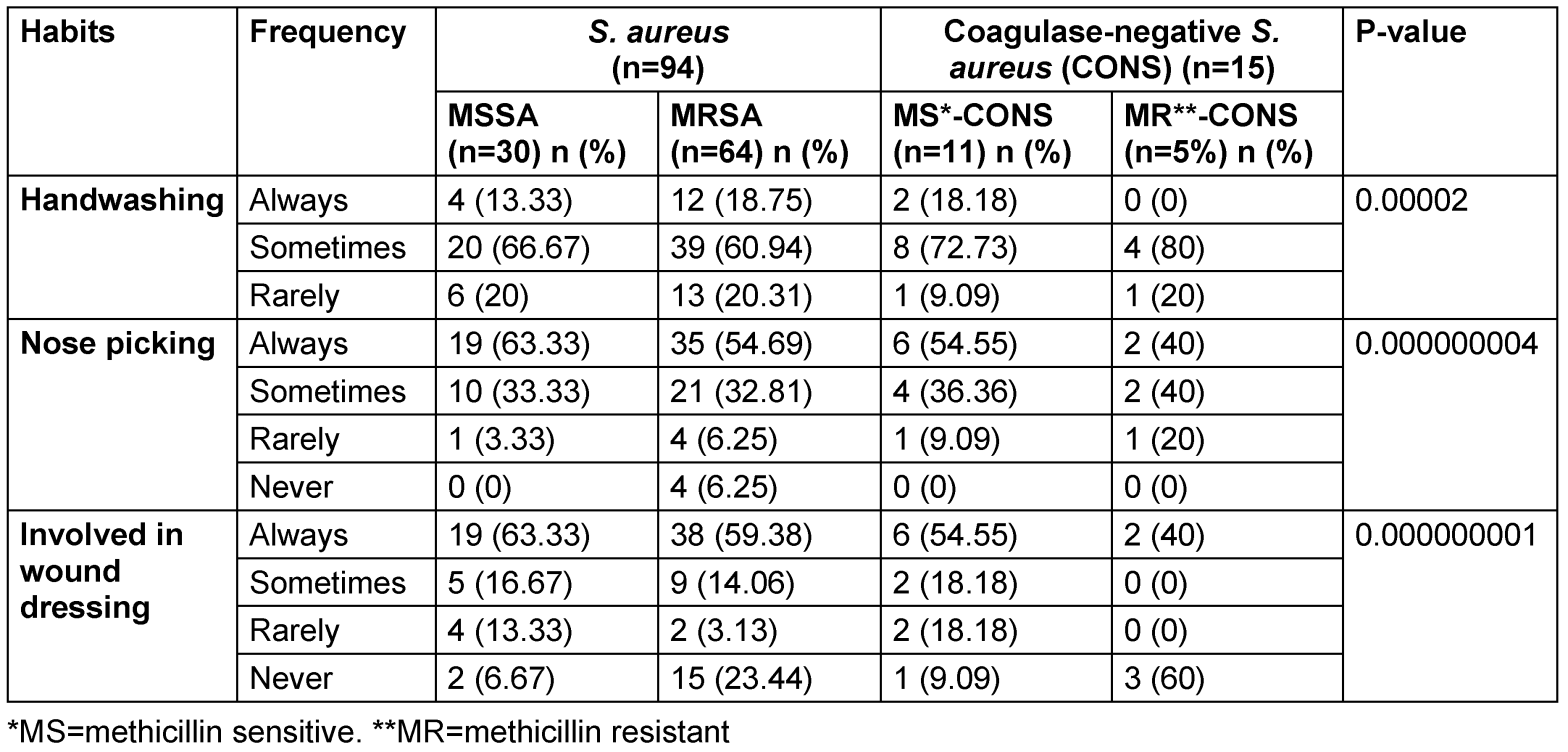
Association between habits and MRSA carriage
